# BCIAUT-P300: A Multi-Session and Multi-Subject Benchmark Dataset on Autism for P300-Based Brain-Computer-Interfaces

**DOI:** 10.3389/fnins.2020.568104

**Published:** 2020-09-18

**Authors:** Marco Simões, Davide Borra, Eduardo Santamaría-Vázquez, GBT-UPM, Mayra Bittencourt-Villalpando, Dominik Krzemiński, Aleksandar Miladinović, Neural_Engineering_Group, Thomas Schmid, Haifeng Zhao, Carlos Amaral, Bruno Direito, Jorge Henriques, Paulo Carvalho, Miguel Castelo-Branco

**Affiliations:** ^1^Coimbra Institute for Biomedical Imaging and Translational Research (CIBIT), Institute of Nuclear Sciences Applied to Health (ICNAS), University of Coimbra, Coimbra, Portugal; ^2^Centre for Informatics and Systems (CISUC), Department of Informatics Engineering, University of Coimbra, Coimbra, Portugal; ^3^Department of Electrical, Electronic and Information Engineering “Guglielmo Marconi” (DEI), University of Bologna, Cesena, Italy; ^4^Grupo de Ingeniería Biomédica, Universidad de Valladolid, Valladolid, Spain; ^5^Centro de Investigación Biomédica en Red, Biomateriales y Nanomedicina, Madrid, Spain; ^6^Biomedical Engineering and Telemedicine Centre, ETSI Telecomunicación, Center for Biomedical Technology, Universidad Politécnica de Madrid, Madrid, Spain; ^7^Department of Neurology, University Medical Center Groningen, University of Groningen, Groningen, Netherlands; ^8^CUBRIC, School of Psychology, Cardiff University, Cardiff, United Kingdom; ^9^Department of Engineering and Architecture, University of Trieste, Trieste, Italy; ^10^Department of Biosciences and Bioengineering, Indian Institute of Technology, Guwahati, India; ^11^Data Science Research Group, School of Computing, University of Kent, Chatham, United Kingdom; ^12^Machine Learning Group, Universität Leipzig, Leipzig, Germany; ^13^The University of Sydney, Camperdown, NSW, Australia

**Keywords:** P300, EEG, benchmark dataset, brain-computer interface, autism spectrum disorder, multi-session, multi-subject

## Abstract

There is a lack of multi-session P300 datasets for Brain-Computer Interfaces (BCI). Publicly available datasets are usually limited by small number of participants with few BCI sessions. In this sense, the lack of large, comprehensive datasets with various individuals and multiple sessions has limited advances in the development of more effective data processing and analysis methods for BCI systems. This is particularly evident to explore the feasibility of deep learning methods that require large datasets. Here we present the BCIAUT-P300 dataset, containing 15 autism spectrum disorder individuals undergoing 7 sessions of P300-based BCI joint-attention training, for a total of 105 sessions. The dataset was used for the 2019 IFMBE Scientific Challenge organized during MEDICON 2019 where, in two phases, teams from all over the world tried to achieve the best possible object-detection accuracy based on the P300 signals. This paper presents the characteristics of the dataset and the approaches followed by the 9 finalist teams during the competition. The winner obtained an average accuracy of 92.3% with a convolutional neural network based on EEGNet. The dataset is now publicly released and stands as a benchmark for future P300-based BCI algorithms based on multiple session data.

## Introduction

A Brain-Computer Interface (BCI) is a system that provides a direct communication between the brain and a computer or external device (Wolpaw and Winter Wolpaw, 2012). In short, it must interpret brain activity and translate it into commands that can be used to control devices or programs, from prosthesis, orthosis, wheelchairs and other robots to a mouse or a keyboard in a controlled computer environment (Bamdad et al., 2015; Chaudhary et al., 2016; McFarland and Wolpaw, 2017). Different types of neuroimaging techniques can be used to implement BCIs, i.e., electroencephalography (EEG), magnetoencephalography (MEG), functional Magnetic Resonance Imaging (fMRI), functional Near-Infrared Spectroscopy (fNIRS), among others (Zou et al., 2019). The most common modality is the EEG, since it provides a portable, inexpensive, non-invasive solution to measure brain activity with high temporal resolution (Sitaram et al., 2007; Bhattacharyya et al., 2017; Deshpande et al., 2017; Zou et al., 2019).

There are several approaches to generate brain signals that can be interpreted and transformed into commands by the BCIs, namely event-related potentials (the most prominent being the P300), steady-state visual evoked potentials (SSVEP) or event-related synchronization/desynchronization (ERS/D) through mental imagery. The P300 approach, first attempted by Farwell and Donchin in the 80s (Farwell and Donchin, 1988), uses an oddball paradigm where an infrequent stimulus of interest is presented in a sequence of frequent stimuli of non-interest. With this paradigm, a positive deflection of the EEG measured in the central and posterior parts of the scalp is observed approximately around 300 ms after the infrequent stimulus of interest is presented (Guo et al., 2019; Riggins and Scott, 2019). The most common application of P300-based BCIs is the speller, where a matrix of letters flashing at different times is presented to the user. An infrequent event occurs due to selective attention to a specific target letter. Thus, a P300 potential is elicited whenever the letter the user is paying attention to flashes, and so the target letter can be identified by a P300 detection algorithm and then transmitted. The use-cases of P300-based BCIs have greatly increased over the past years, from steering a wheelchair (Lopes et al., 2016) to composing music (Pinegger et al., 2017).

Despite the wide range of applications, there are still many challenges facing P300-based BCIs to be used more broadly. Achieving portable and practical BCIs that are easy to setup and fast to calibrate is currently a research line of big interest, since it would favorably help the adoption of this new technology in everyday settings (Amaral et al., 2017; Nakanishi et al., 2019; Zou et al., 2019). However, different issues causing low robustness and reliability should be addressed for these systems to be used in real life. Indeed, often low performance is obtained by BCI models, even in laboratory conditions. The noise sensitivity, non-linearity and non-stationarity characteristics of EEG signals represent critical challenges since these properties depend both on the subject and the environment (Yger et al., 2017). As a consequence of non-stationarity, shifts in EEG signals across trials and sessions occur. Therefore, robust feature extraction techniques are needed to overcome these perturbations on the signals (Raza et al., 2019). Moreover, inter-subject variability, due to anatomical and physiological differences among subjects, also represents an important challenge since it hinders the design of participant-agnostic BCIs. Due to these main challenges (intra- and inter-subject variabilities), most BCIs require time-consuming calibrations to maximize their performance, which makes the creation of one-model-fits-all solutions difficult (Saha and Baumert, 2020).

Nevertheless, the methods used for correctly identifying P300 signals have improved in the last years (Lotte et al., 2018). Traditional decoding algorithms rely on separate feature extraction and classification steps. Commonly used P300 features are based on temporal, time-frequency and spatial domains (Demiralp et al., 2001; Bostanov and Kotchoubey, 2006; Agapov et al., 2016), while Linear Discriminant Analysis (LDA), Support Vector Machine (SVM) and Multi-Layer Perceptron (MLP) are the most prominent classifiers used in P300-based BCI approaches. Some examples of recent improvements over traditional methods are the use of Riemannian geometry (Korczowski et al., 2015) or weightless neural networks (Simões et al., 2019). Recently, deep learning techniques were transposed from the computer vision (LeCun et al., 2015) to the EEG decoding field. Among these new solutions, Convolutional Neural Networks (CNN) and CNNs including recurrent layers - such as Long Short-Term Memories (LSTM) - on top of the convolutional extractor were used (CNN-LSTM) (Craik et al., 2019). A key property of these algorithms is that they automatically learn the relevant features for a given task (i.e., the features are learned from the input data without any *a priori* feature extraction and selection) and finalize the target decoding task in an end-to-end fashion (i.e., without separating these steps). Nevertheless, these approaches pose some challenges: they require many hyper-parameters to be tuned (e.g., number of layers, number of kernels, etc.), they introduce a large number of parameters to be optimized during training (which are also difficult to interpret once trained) and thus, require the use of large datasets to achieve state-of-the-art decoding performance (Lawhern et al., 2018; Craik et al., 2019; Zhang et al., 2019). However, few datasets can be found in the literature matching this last requirement.

To evaluate the efficacy of new methods, authors need to compare their results with current state-of-the-art approaches. One viable approach is to implement both their method and established reference methods and apply all of them to the data of interest. Another option is to use benchmark datasets. Benchmark datasets are publicly available data usually launched in competition events where teams have the same information to start with and try to achieve the best possible result with their methods (Rakotomamonjy and Guigue, 2008). These competitions tend to disclose these datasets afterward, allowing both teams and other researchers to continue developing their methods and publish results that are comparable between them, if researchers recreate the original competition conditions on their attempts. Thus, these datasets provide a common ground for the research areas to assess their methods and improve the state-of-the-art.

One important contributor in this field has been the Berlin Brain-Computer Interface (BBCI) group through the organization of BCI competitions^1^ (Sajda et al., 2003; Blankertz et al., 2004, 2006; Tangermann et al., 2012). The corresponding datasets have been extensively explored and helped significantly the improvement of methods throughout the years (Lotte et al., 2007, 2018). Nevertheless, those datasets were limited in terms of subjects and sessions-per-subject, thus constraining the development of methods highly dependent on multi-session data.

In the scope of the XV Mediterranean Conference in 2019, the International Federation of Medical and Biological Engineering (IFMBE) launched a scientific competition based on a multi-session dataset of P300-based BCI intervention for young adults with autism spectrum disorder (ASD) (Amaral et al., 2018). This intervention was aimed at the rehabilitation of joint-attention, a core developmental skill that is altered in ASD and impacts other skills like language development (Adamson et al., 2019). Joint-attention refers to the ability of following social attentional cues of other people, so one’s attention can be directed by the interlocutor to an external object or event of interest. Amaral et al. (2017) developed an interventional BCI based on P300 signals that uses a virtual environment with a virtual human character and several objects of interest to train the ability of participants to follow the cues of the virtual character to the objects. That system was validated in an interventional pilot study (Amaral et al., 2018) where 15 ASD individuals underwent 7 training sessions with this system. The database resulting from that interventional study supported the 2019 IFMBE scientific challenge and is now made public to the scientific community at https://www.kaggle.com/disbeat/bciaut-p300 (doi: 10.34740/kaggle/dsv/1375326). This paper describes the challenge and corresponding dataset, summarizes the approaches by the competing teams and draws some conclusions from them, challenging the BCI research community to improve the current best performances achieved by the participating teams.

## Materials and Methods

### Experiment Description

#### Overview of the P300-Based BCI System

**FIGURE 1:**
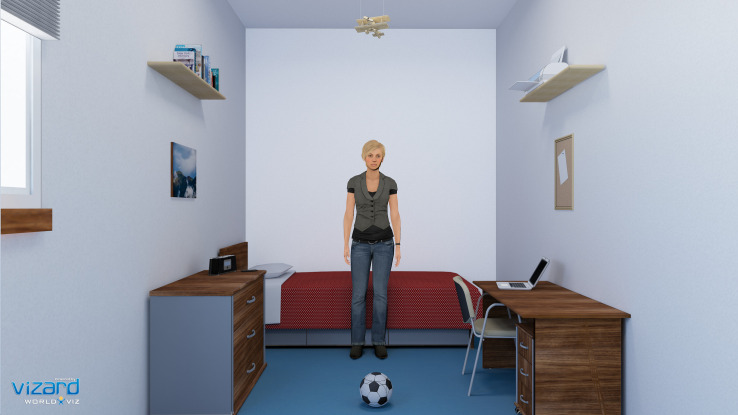
Snapshot of the virtual environment, showing the scenario, the virtual avatar and the objects for joint-attention targets.

The BCI system is composed mainly by two modules: data acquisition module and stimuli presentation module. For the data acquisition module, we used the g.Nautilus system (g.tec medical engineering GmbH, Austria) to record EEG data from 8 active electrodes positioned at C3, Cz, C4, CPz, P3, Pz, P4, POz locations. The reference electrode was placed at the right ear and the ground electrode at AFz location. Sampling rate was set to 250 Hz and data were acquired notch-filtered at 50 Hz and passband-filtered between 2 and 30 Hz. As for the stimuli presentation module, we used the Vizard toolkit to create and display a virtual environment consisting of a bedroom with common type of furniture (shelves, a bed, a table, a chair, and a dresser) and objects (frames, books, lights, a printer, a radio, a ball, a door, a window, and a laptop), as shown in Figure 1. The objects used as stimuli throughout the experiment (and their respective labels) were: 1. books on a shelf, 2. a radio on top of a dresser, 3. a printer on a shelf, 4. a laptop on a table, 5. a ball on the ground, 6. a corkboard on the wall, 7. a wooden plane hanging from the ceiling, and 8. a picture on the wall. The virtual environment was presented via the Oculus Rift Development Kit 2 headset (from Oculus VR).

**FIGURE 2:**
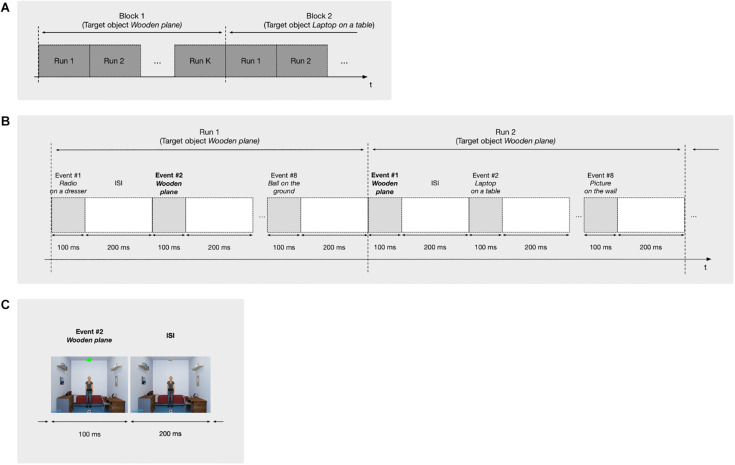
Structure of the paradigm with its subdivisions in blocks, runs and events. **(A)** Structure of the blocks: each block is used to identify a single target object and is composed by K runs. **(B)** Structure of the runs: each run is composed by 8 events, each consisting of the flashing of one of the objects. **(C)** Structure of an event: it consists of the flashing of the corresponding object by 100 ms, followed by an interval of 200 ms.

Each block consists of the user trying to identify one of the objects as the target. For that, *K* runs are repeated. One run is composed by a single flash of each object once for 100 ms at different times and random order, with an Inter-Stimulus Interval (ISI) of 200 ms. Figure 2 provides a schematic for this structure.

#### BCI Session Flow

Fifteen participants performed 7 identical training sessions in different days, the first four on a weekly basis and the last three on a monthly basis. Each training session was divided in two parts: calibration and online phase. Data from calibration and online phases were named in the dataset as train and test data, respectively.

The calibration phase was composed of 20 blocks, each block containing 10 runs. Because we used 10 runs per block, a total of 200 target P300 signals and 1400 non-target signals were acquired at this phase. With these data, the session-specific classifiers were trained for the online phase and the number of runs per block (K) to use on the online phase was defined. K was defined during the online sessions of the clinical trial as the minimum number of runs for which the classifier achieved an accuracy above 80%, in the calibration data.

Regarding the online phase, 50 blocks were taken for each participant using K runs per block. The value of K varied between subjects and sessions, since it was an output of the calibration phase, ranging from 3 to 10.

### Dataset Structure and Contents

The dataset folder structure is organized by subjects, with a folder for each subject named *SBJXX*, with *XX* varying from 01 to 15. Within each subject folder there is a set of folders containing the data from each session, named *SYY*, with *YY* varying from 01 to 07. Each session folder contains a separate folder for the training and testing data, named *Train* and *Test*, respectively. The structure and the contents of train and test folders of each session are described in Box 1.

**Box 1. Dataset Folder Structure.**

SBJ01\

SBJ02\

..

SBJXX\

             S01\

             S02\

..

             SYY\

                         Train\

                         Test\

#### Train folder

• **trainData.mat –** Data from the calibration phase, structured as [*channels* x *epoch* x *event*], epoch corresponding to the data samples from −200 ms to 1000 ms relative to the event stimulus onset (epoch length of 1200 ms; 300 data samples).
• **trainEvents.txt –** One label per line (from 1 to 8), corresponding to the order of the flashed objects.
• **trainTargets.txt –** 1 or 0 per line, indicating if the flashed object was the target or not, respectively.
• **trainLabels.txt –** Label of the target object per line (from 1 to 8), one for each block.

#### Test folder

• **testData.mat –** Data from the online phase, in the same structure as the train data.
• **testEvents.txt –** One label per line (from 1 to 8), corresponding to the order of the flashed objects.
• **testTargets.txt –** 1 or 0 per line, indicating if the flashed object was the target or not, respectively.
• **testLabels.txt –** Label of the target object per line (from 1 to 8), one for each block.
• **runs_per_block.txt –** File containing only one number, corresponding to the number of runs per block used in the online phase (from 3 to 10).

The number of epochs corresponds to # events per run ^∗^ # runs per block ^∗^ # blocks. For the training data, it represents 8 events per run ^∗^ 10 runs per block ^∗^ 20 blocks = 1600 epochs. As for the test data, since the number of runs varies between sessions, the number of epochs varies in consequence, in a total of 8 events per run ^∗^ K runs per block ^∗^ 50 blocks = 400 ^∗^ K epochs.

The channels’ order in the data matrices is C3, Cz, C4, CPz, P3, Pz, P4, POz. The first sample of each epoch corresponds to the time −200 ms relative to the stimulus onset and the last sample to corresponds to the time 996 ms after the stimulus onset (the last sample < 1000 ms), with a sampling rate of 250 Hz, for a total of 300 samples.

### Challenge Structure

For the 2019 IFMBE Scientific Challenge, teams were asked to maximize the P300-based object detection accuracy for the 7 sessions of the 15 ASD participants of the BCIAUT clinical trial. For each session, a train and test set were created, without disclosing the true labels of the test sets. The challenge was divided into two phases with a different number of attempts per phase (Table 1). For *phase I*, sessions 1–3 were provided, without the test labels. At the end of *phase I*, the true test labels of those three sessions were made available to the participants along with the remaining 4 sessions (4–7), the latter without the true test labels (*phase II*). This way, teams could use the true labels of the first three sessions to improve their classifiers, if working with multi-session data. Teams were allowed to submit 5 attempts during phase I and 10 attempts during phase II. The best submission of each team throughout the allowed attempts on each phase was used to rank the teams. The complete dataset (including all true labels) is now available at https://www.kaggle.com/disbeat/bciaut-p300 (doi: 10.34740/kaggle/dsv/1375326).

**TABLE 1:** Timetable and number of attempts for the two phases of the competition.

**Phase**	**Start Date**	**End Date**	**Number of Attempts**
*Phase I*	01-03-2019 10:00	15-05-2019 23:59	5
*Phase II*	20-05-2019 10:00	30-06-2019 23:59	10

### Submissions and Approaches

Fourteen teams participated in phase I of the competition, while 9 teams participated in phase II and concluded the challenge. The results shown in this manuscript refer to the phase II of the competition. The performance metric used to compare the performance of contesting teams was the target object detection accuracy, computed as the ratio between the number of correct predicted blocks and the total number of blocks to decode. Based on the average target object accuracy across subjects and sessions, the approaches proposed by each team were ranked up.

The following list of IDs reflects the final ranking of the competition:

• ***ID-1***: DB, Silvia Fantozzi and Elisa Magosso (Borra et al., 2020a).
• ***ID-2***: Eduardo Santamaría-Vázquez, Víctor Martínez-Cagigal, Javier Gomez-Pilar and Roberto Hornero (Santamaría-Vázquez et al., 2020).
• ***ID-3***: Lucia de Arancibia, Patricia Sánchez-González, Enrique J. Gómez, M. Elena Hernando and Ignacio Oropesa (de Arancibia et al., 2020).
• ***ID-4***: MB-V and Natasha M. Maurits (Bittencourt-Villalpando and Maurits, 2020).
• ***ID-5***: DK, Sebastian Michelmann, Matthias Treder and Lorena Santamaria (Krzemiński et al., 2020).
• ***ID-6***: AM, Miloš Ajćević, Giulia Silveri, Gaia Ciacchi, Giulietta Morra, Joanna Jarmolowska, Piero Paolo Battaglini and Agostino Accardo (Miladinović et al., 2020).
• ***ID-7***: Bipra Chatterjee, Ramaswamy Palaniappan and Cota Navin Gupta (Chatterjee et al., 2020).
• ***ID-8***: V. Sophie Adama, Schindler Benjamin and TS (Adama et al., 2020).
• ***ID-9***: HZ, Shiduo Yu, Joseph Prinable, Alistair McEwan and Petra Karlsson (Zhao et al., 2020).

**FIGURE 3:**
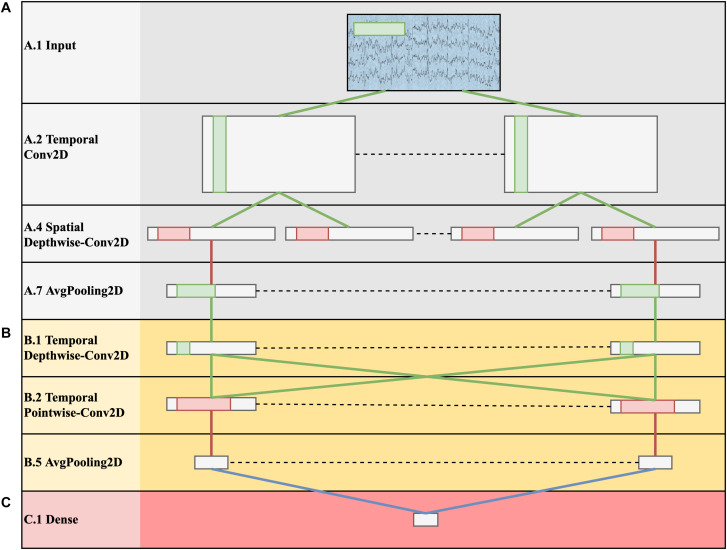
Architecture schematization of the winning solution *ID-1* based on *EEGNet*. The represented shapes correspond to the output of each layer. Green lines represent convolutional connections, red lines pooling connections, and blue lines dense connections. The CNN is composed by a temporal and spatial feature extractor **(A)**, a summary feature extractor **(B)** and a classification module **(C)**.

For each team, a brief description of the proposed methodology is reported:

• ***ID-1***: Epochs were extracted between −100–1000 ms, and the signals were downsampled to 128 Hz. The decoding solution was based on a CNN performing classification at the level of single trial (EEG response to a single stimulus, without averaging). The input was a 2-D representation composed by the EEG channels along one dimension (spatial dimension) and time steps along the other dimension (temporal dimension). The CNN was an adaptation of EEGNet (Lawhern et al., 2018) trained to discriminate between P300 and non-P300 classes. In this CNN design, depthwise and pointwise convolutions are used to keep the number of trainable parameters limited. The architecture in its fundamental subnetworks and main connections between neurons is displayed in Figure 3. Furthermore, a detailed description of these subnetworks including the main hyper-parameters, output activation shapes and number of trainable parameters introduced is reported in Table 2. The CNN is composed by 3 main subnetworks (here labeled as A, B, C), performing different operations on the input. These include a temporal and spatial feature extractor (Figure 3A) that learns meaningful temporal and spatial filters, a summary feature extractor (Figure 3B) that learns to extract temporal summaries for each feature map of the subnetwork A individually; and a classification module (Figure 3C) that finalizes the classification task based on the output of the subnetwork B. The obtained single-trial probabilities were then averaged together across runs related to a specific object belonging to each block, and then the object with maximum average probability was selected, solving the target 8-way classification task. Different intra-subject training strategies were explored, including inter-session (i.e., training subject-specific classifiers) and intra-session (i.e., training session-specific classifiers) training strategies. The top-performing solution of *ID-1* was the one adopting a subject-wise inter-session strategy. The code of the CNN and the weights of the trained models are available at https://github.com/ddavidebb/IFMBE2019Challenge-BCIAUT-P300.

**TABLE 2:** Architecture design inspired from EEGNet and adopted in *ID-1*.

**Subnet.**	**Layer ID**	**Layer**	**Hyper-parameters**	**# pars**	**Output shape**	**Activation**
A	A.1	Input		0	(1,8,140)	
	A.2	Temporal Conv2D	K = 8, F = (1,65), *P* = (0,32)	520	(8,8,140)	Linear
	A.3	BatchNorm2D		16	(8,8,140)	
	A.4	Spatial Depthwise-Conv2D*	D = 2, K = 16, F = (8,1), *P* = (0,0)	128	(16,1,140)	Linear
	A.5	BatchNorm2D		32	(16,1,140)	
	A.6	Activation		0	(16,1,140)	Exponential Linear Units (ELU)
	A.7	AvgPooling2D	F = (1,4)	0	(16,1,35)	
	A.8	Dropout	*p* = 0.25	0	(16,1,35)	
B	B.1	Temporal Depthwise-Conv2D	D = 1, K = 16, F = (1,17), *P* = (0,8)	272	(16,1,35)	Linear
	B.2	Temporal Pointwise-Conv2D	K = 16, F = (1,1), *P* = (0,0)	256	(16,1,35)	Linear
	B.3	BatchNorm2D		32	(16,1,35)	
	B.4	Activation		0	(16,1,35)	ELU
	B.5	AvgPooling2D		0	(16,1,4)	
	B.6	Dropout	*p* = 0.25	0	(16,1,4)	
C	C.1	Dense	*N* = 2	130	(2)	Linear
	C.2	Activation		0	(2)	Softmax

K and F are the number and the size of the kernels, respectively. P is the padding size, D the depth multiplier, N the number of neurons in the dense layer and finally p the dropout rate. Light-gray denote layers with trainable parameters. The total number of trainable parameters is 1386. *Unitary kernel max-norm constraint.

• ***ID-2***: EEG signals were epoched between 0–1000 ms, applying a baseline (−200-0 ms) normalization. The input representation is the same as in ***ID-1***. The task was faced as a 2-way classification decoding P300 and non-P300 classes for each trial adopting an adaptation of the CNN proposed by Manor et al. (Manor and Geva, 2015), a CNN-LSTM and a CNN-BLSTM. Furthermore, these deep learning architectures were compared with a more traditional machine learning pipeline including SWLDA. The top-performing algorithm proposed by ***ID-2*** was CNN-BLSTM. This network was composed of one convolutional layer 1-D that extracts spatio-temporal patterns on the input, two bidirectional LSTM layers and one dense layer. The single-trial probabilities were averaged to obtain object-level probabilities as in ***ID-1***. An intra-subject and inter-session training strategy was adopted, training subject-specific classifiers. The code of the models and the weights of the trained models are available at https://github.com/esantamariavazquez/IFMBE2019Challenge-BCIAUT-P300.
• ***ID-3***: EEG signals related to a specific object were averaged across trials of the same block. Feature extraction was based on temporal and time-frequency parameters. Temporal features were extracted in epochs between 0–1000 ms by downsampling the signals with a decimation factor of 10. In addition to temporal features, features based on continuous wavelet transform (CWT) were extracted from epochs between 200–712 ms. The t-CWT was computed based on a Mexican Hat wavelet on scales corresponding to the delta (0.5–4 Hz) and theta (4–8 Hz) bands (Demiralp et al., 2001; Bostanov and Kotchoubey, 2006). These temporal and time-scale features were concatenated across channels in a single vector. Principal component analysis (PCA) was applied for feature dimensionality reduction, which resulted in a final vector of 120 features. A comparison of different combinations of linear and non-linear machine learning approaches was performed. More specifically, linear discriminant analysis (LDA) and support vector machines with linear kernel (LSVM), and a more complex support vector machine with radial kernel (RSVM) were employed. The object whose corresponding signals yielded a higher probability of containing a P300 event was chosen as predicted target object of the block. In addition, the effect on the accuracy of the number of EEG events averaged was studied. An inter-session training strategy was adopted, comparing both subject-specific and inter-subject classifiers, as well as the use of oversampling and boosting techniques to account for class imbalance. LDA outperformed the other classifiers and was used to classify the target object. Best results were obtained for > 3 events averaged. Training subject-specific classifiers yielded the best performance. Oversampling and boosting did not improve the final performance of the classifiers. The developed code and trained models are available at: http://dev.gbt.tfo.upm.es/ioropesa/ifmbe-scientific-challenge-competition—detection-of-p300/tree/master.
• ***ID-4:*** The approach consisted of the adaptation and parameter optimization of an SVM-based algorithm that was previously developed for a 4-choice BCI (Bittencourt-Villalpando and Maurits, 2018) for target identification. During the first phase of the challenge, the original algorithm was adapted for 8 choices and the pre-processing parameters were defined as follows. First, temporal features were extracted in epochs between 0–1000 ms following each event onset and all channels were concatenated in a single feature vector per event for each participant and session. Then, feature vectors containing EEG signals from target events were pseudo-randomly averaged across blocks belonging to the same session for noise reduction. During the second phase of the challenge, an intra-subject and intra-session training strategy was developed, augmenting the dataset with other sessions’ signals, and artificially increasing the number of targets per session by adapting the pseudorandom averaging procedure. Eight parameters related to data augmentation and SVM input parameters were optimized throughout the 9 initial attempts and then compared in terms of accuracy. The parameters’ description and settings per attempt are detailed in Bittencourt-Villalpando and Maurits (2020). In the last attempt, the best performing parameter setting was selected, resulting in a customized solution per participant and per session.
• ***ID-5:*** This solution exploited Riemannian framework for EEG signal decoding (Korczowski et al., 2015). The approach was computationally efficient and recently outperformed other common state-of-the-art approaches (Barachant et al., 2010). The Riemannian framework was combined with the ensemble learning. The idea was to build upon many “weak” (under-performing) classifiers and then combine their outcomes to improve the performance of the final model. The ensemble of 8 different data features was constructed by combining 2 different band-pass filters (1–20 Hz or 1–8 Hz), 2 trial lengths (from −200 to 1000 ms or from 0 to 600 ms) and 3 different subsets of electrodes (all, central or posterior only electrodes). Then, the ERP prototypes were created by calculating the ERP for each channel. Next, the regularized covariance matrices of a single trial concatenated with the prototype were computed and the resultant matrices were projected into the tangent space of a reference matrix. Fisher Geodesic Discriminant Analysis (FGDA) was used to project the matrices to a lower-dimensional discriminative subspace. The resultant projections were flattened to vectors and used as the features to the ensemble learning algorithm comprising 400 LDA classifiers. The output probability was aggregated across trials belonging to each object to decode the target per each block. An intra-subject and intra-session training strategy was adopted. The developed code is available at https://github.com/dokato/bci-challange.
• ***ID-6:*** The windows mean approach was used to obtain the temporal features on each trial. These were computed for each electrode on 50 ms windows without overlap from 100–1000 ms. Bayesian logistic regression with automatic relevance determination (VB-ARD) (Drugowitsch, 2013) was used to classify the P300 event on each trial. The method has an advantage over other regularization techniques which need a separate validation set to eliminate irrelevant features. Besides, this approach generates a posterior distribution enabling the authors to model the varying-intercept sparse feature model. The modeling applied in this approach is similar to the one proposed by Bishop (2006) with a variation of Automatic Relevance Determination (ARD) that instead of using type-II maximum likelihood (MacKay, 1992), applies full Bayesian treatment (Drugowitsch, 2013). The primary generative model matches the one employed in Bishop (2006), and the prior is selected to be non-informative, modeled by a conjugate Gamma distribution (Drugowitsch, 2013). This makes the model parameter-free and easy to use without deep knowledge in the data science domain. The advantage of this methodology is that obtained distribution allows the authors to find the inverse of the predictors’ covariance matrix (precision matrix) and apply Automatic Relevance Determination (ARD) that assigns an individual hyper-prior to each regression coefficient separately determining their relevance and produces for each trial a class-belonging probability. Lastly, single-trial probabilities were averaged together across trials for each object belonging and the one with maximum average probability was selected. In this method, an intra-subject and intra-session training strategy was performed. The demo code is available at https://github.com/miladinovic/BCILabTS under subfolder *userscripts*.
• ***ID-7:*** Whole signals were used (−200–1000 ms) and the pre-stimulus mean (−200-0 ms) was removed. Signals were filtered between 2–12 Hz and the filtered signals were downsampled 10-times. Then, these downsampled electrode signals were normalized epoch-wise in the range −100–1000 ms. These temporal features were used to classify the P300 event for each trial with BLDA, RUSBoost and CNN. The best performing classifier for each subject was used (subject-specific classifier). Then, a majority voting was done to determine the target object within each specific block. An intra-subject and inter-session training strategy was performed.
• ***ID-8:*** EEG signals were averaged across trials related to a specific object belonging to each block. Temporal features were extracted for each electrode by averaging for each time window from 200–450 ms and decimating the output with a factor of 12. In addition, Pearson’s correlation coefficient was computed for each electrode between the time window of interest and the time window preceding stimulus presentation (−200-0 ms). These temporal features and correlation coefficients were concatenated across channels in a single feature vector. An inter-subject and inter-session training strategy was performed, by which a variety of competing supervised learning techniques (decision tree, random forest, SVM, MLP) were trained to classify the target object within each block. From those, MLP performed best on the given data.
• ***ID-9:*** Epochs were extracted from 0–600 ms. An additional 20 Hz low-pass filter was applied to the original data. In addition, a custom filter was designed to address each subject- and session-specific noise features. The temporal features were selected using a linear support vector regression as a pre-selector for features in the data. A comparison between linear and non-linear methods was performed, using SVM, LDA, 1D 4-layer CNN, 1-layer LSTM. LDA was the top-performing classification algorithm for *ID-9* and was used to classify the P300 event for each trial. Then, the label that appeared most times within each block was the target object to decode. An intra-subject and intra-session training strategy was adopted. The code is available at https://github.com/hyphenzhao/MEDICON2019ScientificChallenge.

A summary of the top-performing method of each team adopted for the challenge is shown in Table 3.

**TABLE 3:** Summary of the best-performing algorithm of each team developed for the challenge.

**ID #**	**acc. (%)**	**Pre-processing**	**Methodology**	**Post-processing**	**Training strategy**	**Framework**
*ID-1*	92.3 ± 1.8	• Epochs from -100 to 1000 ms • Downsampling to128 Hz	• CNN based on EEGNet (Lawhern et al., 2018)	• Average probability across runs within a specific block • Decoding of the target object as the object with maximum average probability	• Intra-subject and inter-session	• Python with PyTorch
*ID-2*	84.3 ± 3.2	• Epochs from 0 to 1000 ms • Baseline normalization from -200 to 0 ms	• CNN-BLSTM	• Average probability across runs within a specific block • Decoding of the target object as the object with maximum average probability	• Intra-subject and inter-session	• Python with Scikit-learn and Keras
*ID-3*	82.0 ± 2.5	• Temporal features: ° Ensemble averaging per block ° Temporal epoching from 0 to1000 ms. ° Moving-average downsampling• CWT features: ° Temporal epoching from 200 to 712 ms∘ Most differential points computed with t-Student (t-CWT)	• Temporal features concat (200 features)• Computation of the t-CWT (Bostanov and Kotchoubey, 2006) based on Mexican Hat wavelet (128 points per channel) and CWT features concat. (1024 features)• Feature reduction based onyh PCA (120 features)• LDA	• The object whose corresponding signals yield a higher probability of containing a P300 was chosen as predicted target object of the block	• Intra-subject and inter-session	• MATLAB with Statistics and Machine Learning Toolbox and Signal Processing Toolbox
*ID-4*	81.5 ± 2.6	• Epochs from 0 to 1000 ms• Pseudorandom• averaging of ERP segments.	• Feature vector with 2000 elements per ERP (concat. of 8 channels*250 elements)• SVM	• The feature vectors were sorted according to the event (flashed object, from 1 to 8)• All runs per block were averaged, per event• The predicted target corresponds to the event with the highest score.	• Intra-subject and intra-session• Data augmentation with other sessions’ signals and with pseudorandom averaging	• MATLAB with Statistics and Machine Learning Toolbox 2017.
*ID-5*	81.2 ± 2.1	• Band-pass filtering with two different filters (1–20 Hz or 1–8 Hz) and two variations of trial length (whole signal or the first 600 ms after stimuli onset) • Three subsets of electrodes were chosen (all, central or posterior electrodes)	• ERP prototypes were created by calculating the ERP for each channel • Regularized covariance matrices of a single trial signal concatenated with prototype were calculated • The resultant covariance matrices were projected into the tangent space of a reference matrix • FGDA was used to project the matrices in tangent space to a lower-dimensional discriminative subspace. These were used as features. • Ensemble of 400 LDA classifiers (taking 40% of data samples and 60% of features) operated on ensemble of signal preprocessed in 8 different combinations	• Aggregated probability of trial belonging to each of the classes.	• Intra-subject and intra-session	• MATLAB
*ID-6*	80.3 ± 2.2	• Epochs from 100 to 1000 ms	• Temporal features computed on 50 ms windows, without overlap, producing 18 features per channel for each event • VB-ARD	• Average probability across runs within a specific block • Decoding of the target object as the object with maximum average probability	• Intra-subject and intra-session	• MATLAB • BCILAB
*ID-7*	76.3 ± 2.9	• Epochs from −200–1000 ms • Pre-stimulus mean (−200-0 ms) was removed. • Band-pass filtering 2–12 Hz • Normalization epochwise to the interval [−1,1]	• Temporal features were extracted by downsampling with a factor of 10 the normalized and filtered signals • Three classifiers were trained and tested: ° BLDA ° RUSBoost ° CNN	• The best performing classifier for each subject was used • Majority voting within each run to determine which flash has been classified as target maximum number of time and that was predicted as target for that particular run	• Intra-subject and inter-session	• MATLAB with Classification App RUSBoosted Trees
*ID-8*	70.0 ± 3.8	• Averaging of EEG signals across trials related to a specific object within each block	• Temporal features [based on (Krusienski et al., 2006)]: averaging within windows from 200–450 ms; 56 features per channel (448 total) • Pearson’s correlation coefficients: coefficients were computed between the time window of interest and the time window preceding stimulus presentation (-200-0 ms); 8 features per channel (64 total) • Concatenation of temporal and Pearson’s coefficients across channels in a single feature vector MLP	• -	• Inter-subject and inter-session	• MATLAB (pre-processing) • Python with Scikit-learn (main algorithm)
*ID-9*	67.2 ± 3.3	• Epochs from 0–600 ms • Low-pass filter 20 Hz • Custom filter to address each subject- and session-specific noise features deduced from non-target epochs	• Linear support vector regression as feature pre-selector • LDA	• The label that appeared most times within each block was the target object to decode	• Intra-subject and intra-session	• Python with Scikit-learn

### Statistical Analysis

For each team, the best-performing solution proposed among the phase II attempts – in terms of target object accuracy averaged across subjects and sessions – was selected for analysis and the algorithms were then ranked up based on this average score. Furthermore, the metrics scored by algorithms *ID-2:9* were compared with the winning algorithm (*ID-1*) using Wilcoxon signed-rank tests. To correct for multiple tests, a false discovery rate correction at 5% using the Benjamini-Hochberg procedure (Benjamini and Hochberg, 1995) was applied and the corrected *p*-values are reported.

## Results

In Tables 4, 5 the accuracies of the proposed approaches are shown, describing the decoding variability across subjects and recording sessions. In particular, Table 4 reports for each subject the average target object accuracy across sessions (i.e., performance at the level of single subjects), while Table 5 reports for each session the average target object accuracy across subjects (i.e., performance at the level of single session).

**TABLE 4:** Performance at the level of single subject as represented by the average target object accuracies of the best approach proposed by each team.

**ID #**	**Accuracy at the level of single subject (%)**																
	**1**	**2**	**3**	**4**	**5**	**6**	**7**	**8**	**9**	**10**	**11**	**12**	**13**	**14**	**15**	**acc (mean ± SEM)**	***p*-value**
*ID-1*	81	100	86	96	93.5	96	96.5	100	90.5	98	94	84.5	86.5	81.5	100	92.3 ± 1.8	−
*ID-2*	56	98	67.5	96	80	88	86.5	99	82	93	87.5	80	81	71.5	98.5	84.3 ± 3.2	0.0010
*ID-3*	73	95	71	91	82.5	86	85	91.5	68.5	88.5	86	80.5	60	84	87.5	82.0 ± 2.5	0.0009
*ID-4*	64.5	92	68	94.5	84	86	81.5	94	71	87	87	82	66	77	88	81.5 ± 2.6	0.0009
*ID-5*	69	91	67	88.5	79.5	82.5	83	95	82.5	81.5	85.5	79	69	78	87.5	81.2 ± 2.1	0.0009
*ID-6*	68	91.5	71.5	92.5	80	84	79	94.5	73.5	82.5	84	78.5	68.5	71.5	85.5	80.3 ± 2.2	0.0009
*ID-7*	54	93	62.5	90	73	85.5	76	88.5	71	78	80.5	78	65	65	84.5	76.3 ± 2.9	0.0009
*ID-8*	48	84	58	69	69.5	52	84	94	72	87.5	77	64	50	56.5	84.5	70.0 ± 3.8	0.0009
*ID-9*	46	85	53	77	65	66.5	67.5	89	57	72	73.5	73	59.5	47	77.5	67.2 ± 3.3	0.0009

*The best decoding performance for each subject is colored with light-gray. The mean accuracy (acc) and its standard error (SEM) are reported. Wilcoxon signed-rank test was used to compare ID-1 with ID-2:9 and the corrected *p*-values for multiple tests are reported.*

**TABLE 5:** Performance at the level of single session as represented by average target object accuracies across subjects of the best approach proposed by each team.

**ID #**	**Accuracy at the level of single session (%)**							
	**4**		**5**		**6**		**7**	
	**acc (mean ± SEM)**	***p*-value**	**acc (mean ± SEM)**	***p*-value**	**acc (mean ± SEM)**	***p*-value**	**acc (mean ± SEM)**	***p*-value**
*ID-1*	92.8 ± 2.4	−	90.4 ± 3.5	−	94.8 ± 1.8	−	91.1 ± 3.0	−
*ID-2*	85.1 ± 3.1	0.0044	82.0 ± 5.5	0.0026	90.5 ± 2.6	0.0082	79.6 ± 5.6	0.0026
*ID-3*	81.5 ± 3.3	0.0023	82.0 ± 4.4	0.0062	84.3 ± 2.6	0.0025	80.3 ± 3.7	0.0015
*ID-4*	80.3 ± 3.0	0.0015	80.7 ± 4.4	0.0037	84.9 ± 2.6	0.0015	80.1 ± 4.2	0.0020
*ID-5*	79.9 ± 3.3	0.0013	78.4 ± 4.2	0.0015	85.1 ± 2.4	0.0013	81.6 ± 4.2	0.0032
*ID-6*	78.1 ± 3.6	0.0015	79.6 ± 4.0	0.0017	83.6 ± 2.6	0.0013	80.0 ± 3.7	0.0013
*ID-7*	75.2 ± 3.8	0.0013	72.8 ± 4.9	0.0013	80.3 ± 2.6	0.0013	76.9 ± 3.6	0.0013
*ID-8*	70.5 ± 4.3	0.0013	70.3 ± 6.0	0.0013	72.7 ± 3.8	0.0013	66.5 ± 5.7	0.0013
*ID-9*	64.8 ± 4.3	0.0013	66.9 ± 4.5	0.0013	69.3 ± 4.2	0.0013	67.9 ± 5.4	0.0013

*The best decoding performance for each recording session is colored with light-gray. The mean accuracy (acc) and its standard error (SEM) are reported. Wilcoxon signed-rank test was used to compare *ID-1* with *ID-2:9* and the corrected *p*-values for multiple tests are reported.*

Averaging across sessions and across subjects, ***ID-1*** significantly outperformed the other approaches, with less variability across subjects and sessions. Looking at the performance at the level of subjects, ***ID-1*** provided the best performance metric for 14 out of 15 subjects (for subject #4, ***ID-2*** provided a top-performance across the proposed solutions too), while ***ID-3*** provided the best performance metric for 1 out of 15 subjects (subject #14).

Averaging across subjects, ***ID-1*** significantly outperformed the other approaches within each recording session, with less variability across subjects and providing an average performance above 90% for all the phase II sessions.

## Discussion

In this study, a large multi-session and multi-subject dataset acquired during a P300-based BCI intervention for young adults with ASD was presented. The evolution and the practical application of deep learning solutions for EEG decoding depend on the availability of large multi-subject datasets. Furthermore, the lack of multi-session datasets hinders the design of reliable algorithms across recording sessions. Thus, the described dataset represents a multi-session collection of signals that can be used as a benchmark to design accurate and reliable data-hungry algorithms, such as deep learning solutions, for P300 decoding tasks.

In fact, the richness of the dataset enabled the use of deep learning approaches in the context of the competition. Among the proposed algorithms, a deep learning solution based on a lightweight CNN (see ***ID-1*** in Section “Submissions and Approaches”) outperformed both a CNN-BLSTM (*p* = 0.001, across subjects and sessions, see Table 4, ***ID-2***) and more traditional machine-learning solutions (*p* < 0.001, across subjects and sessions, see Table 4). Furthermore, this was found also for single session recordings (*p* < 0.005 when comparing ***ID-1*** with other solutions, see Table 5), with average metrics above 90% (far above the chance level of 12.5%). The best non-deep learning solution adopted temporal and CWT features, alongside with PCA for dimensionality reduction and LDA for classification (see ***ID-3*** in Section “Submissions and Approaches”). The training strategies performed in the approaches ***ID-1*:*3*** were both intra-subject and inter-session. In particular for the winning solution, from the experiments between inter-session and intra-session trainings performed by ***ID-1***, better results were found using all the session signals during the optimization.

When using deep learning approaches with EEG signals, the input representation and the design of spatio-temporal convolutions is not trivial and need to be addressed. Regarding the input representation, the time series are related to electrodes placed on a 3D surface. Typically, EEG signals can be represented in three different ways to feed the input layer of a neural network (Lawhern et al., 2018):

a. Using the original representation of all the available electrode signals to design a 2D representation where EEG channels are reported along one dimension (spatial dimension) and time steps along the other dimension (temporal dimension).
b. Using a transformed representation (e.g., time-frequency decomposition) of all the available electrodes.
c. Using a representation as in (b) with a subset of electrodes.

Among these representations, the first one is preferred since a representation like (b) generally increases the dimensionality (Lawhern et al., 2018), leading to more trainable parameters and, thus, to the need of more data or an increased regularization. Furthermore, several hyper-parameters are introduced depending on the transformation applied. Lastly, representations like (c) share the main disadvantages of (b) with an additional needing of *a priori* knowledge about the more relevant subset of electrodes to choose. Therefore, representations that respect the scheme (a) are a good compromise between input dimensionality and capability to learn more general EEG features on all the electrode signals (Lawhern et al., 2018). Among the best-performing solutions in this competition, ***ID-1*** and ***ID-2*** adopted the first input representation scheme.

Regarding the design of spatio-temporal convolutions, depending on the information processing in the convolutional module, three different solutions can be designed starting from the input layer:

i. The temporal filtering is performed at first and then the spatial filtering.
ii. The spatial filtering is performed at first and then the temporal filtering.
iii. Mixed spatio-temporal filtering.

The CNN adopted by *ID-1* used the convolutions ordering as in (i), while the CNN-BLSTM adopted by *ID-2* as in (iii). Furthermore, among the solutions proposed by *ID-2*, there was a CNN based on Manor and Geva (2015) adopting a convolution ordering as in (ii). Thus, in this competition, the solutions based on convolution ordering as in (i) outperformed the solutions following (ii) and (iii) designs.

In addition, the layers of the neural network need to be carefully designed to keep control the number of trainable parameters and thus, to avoid overfitting when handling a limited collection of training signals. To this aim, architectures like EEGNet (Lawhern et al., 2018) were proposed including optimized convolutions, such as depthwise and separable convolutions (Chollet, 2016). The CNN adapted in ***ID-1*** was inspired from Lawhern et al. (2018) and introduced only 1386 trainable parameters, while the CNN-BLSTM designed by ***ID-2*** introduced 10113 parameters. Lastly, among the solutions proposed by ***ID-2*** (different from the best-performing algorithm of ***ID-2***), a CNN based on Manor et al. (Manor and Geva, 2015) introduced 37428963 parameters. Therefore, in this competition the use of a lightweight architecture to solve the target P300 decoding task was beneficial. This result is in line with the recent growth of interest in the design of optimized layers in CNNs for EEG decoding as proposed by Zhao et al. (2019) and Borra et al. (2020b).

The **BCIAUT-P300** dataset presents rare characteristics which reinforce its potentialities to work as a benchmark for P300-based BCI methods: 1) the multi-subject dimension, with 15 participants undergoing the same procedure, enable the possibility of developing inter-subject methods for generalized off-the-shelf applications; 2) the multi-session dimension, since each subject repeated the same training task 7 times in different weeks, enables the study of stability and reliability of subject-specific BCI methods throughout time, and even the inclusion of reinforcement learning strategies by approaching the sessions gradually; and 3) the ASD clinical dimension, since real-life BCI applications on ASD patients pose several challenges, this dataset provide a test bench for data quality and artifactual EEG data on ASD population that new projects can use to validate its models before approaching the clinical patients directly.

## Conclusion

This paper presented the BCIAUT-P300 dataset which combines multi-session and multi-subject data of 15 ASD participants using a P300-based BCI for training joint-attention skills. The dataset was used on the IFMBE scientific competition where 9 teams from around the world reach the final phase and presented their methods, which were briefly presented here. Overall, deep learning methods were able to overcome the more traditional machine learning approaches, with the best method obtaining an average accuracy of 92.3%. Future studies should address the multiple dimensions of the dataset to reduce training times while improving accuracy.

## Members of the GBT-UPM and Neural_Engineering_Group

GBT-UPM: Lucía de Arancibia, Patricia Sánchez-González, Enrique J. Gómez, M. Elena Hernando, and Ignacio Oropesa.

Neural_Engineering_Group: Bipra Chatterjee, Ramaswamy Palaniappan, and Cota Navin Gupta.

## Data Availability Statement

The datasets presented in this study can be found in online repositories. The names of the repository/repositories and accession number(s) can be found below: https://www.kaggle.com/disbeat/bciaut-p300 (doi: 10.34740/kaggle/dsv/1375326).

## Ethics Statement

The studies involving human participants were reviewed and approved by CEIC—Comissão de Ética para a Investigação Clínica (Portuguese Ethics Committee for Clinical Research). Written informed consent to participate in this study was provided by the participants’ legal guardian/next of kin.

## Author Contributions

MS organized the challenge, the dataset and partially wrote most of the sections of the document along with DB. DB also performed the statistical analysis. DB, ES-V, GBT-UPM, MB-V, DK, AM, NEG, TS, and HZ wrote the ID-1:9 descriptions, in that order. CA was responsible for the BCI system development and data collection and, along with MS, BD, and JH coordinated the IFMBE Scientific Challenge. PC supervised the IFMBE Scientific Challenge and MC-B was the PI of the BCIAUT clinical trial, providing scientific guidance to all the process. All authors reviewed and made significant contributions to the final document.

## Conflict of Interest

The authors declare that the research was conducted in the absence of any commercial or financial relationships that could be construed as a potential conflict of interest.

## Footnotes

1 http://www.bbci.de/activities#competition
